# microRNAs Involved in Regulating Spontaneous Recovery in Embolic Stroke Model

**DOI:** 10.1371/journal.pone.0066393

**Published:** 2013-06-18

**Authors:** Fu Jia Liu, Kai Ying Lim, Prameet Kaur, Sugunavathi Sepramaniam, Arunmozhiarasi Armugam, Peter Tsun Hon Wong, Kandiah Jeyaseelan

**Affiliations:** 1 Department of Biochemistry, Center for Translational Medicine, Yong Loo Lin School of Medicine, National University Health System, National University of Singapore, Singapore, Singapore; 2 Department of Pharmacology, Center for Translational Medicine, Yong Loo Lin School of Medicine, National University Health System, National University of Singapore, Singapore, Singapore; 3 Department of Anatomy and Developmental Biology, School of Biomedical Sciences, Faculty of Medicine, Nursing and Health Sciences, Monash University, Clayton, Victoria, Australia; School of Pharmacy, Texas Tech University HSC, United States of America

## Abstract

To date, miRNA expression studies on cerebral ischemia in both human and animal models have focused mainly on acute phase of ischemic stroke. In this study, we present the roles played by microRNAs in the spontaneous recovery phases in cerebral ischemia using rodent stroke models. Brain tissues were harvested at different reperfusion time points ranging from 0–168 hrs after middle cerebral artery occlusion using homologous emboli. MiRNA and mRNA expression profiles were investigated by microarray followed by multiple statistical analysis. Candidate transcripts were also validated by quantitative RT-PCR. Three specific groups of miRNAs were observed among a total of 346 differentially expressed miRNAs. miRNAs, miR-21, -142-3p, -142-5p, and -146a displayed significant upregulation during stroke recovery (48 hrs to 168 hrs) compared with those during acute phases (0 hrs to 24 hrs). On the other hand, an opposite trend was observed in the expression of miR-196a/b/c, -224 and -324-3p. Interestingly, miR-206, -290, -291a-5p and -30c-1*, positively correlated with the infarct sizes, with an initial increase up to 24hrs followed by a gradual decrease from 48 hrs to 168 hrs (R = 0.95). Taken together with the expression levels of corresponding mRNA targets, we have also found that Hedgehog, Notch, Wnt and TGF-β signaling pathways could play significant roles in stroke recovery and especially in neuronal repair.

## Introduction

Stroke is one of the leading causes of death and adult disability worldwide. The possible strategies for ischemic stroke treatment include intravenous thrombolysis (rt-PA), neuroprotection and neuronal repair. Although rt-PA has been the only approved treatment for ischemic stroke, the risk of haemorrhage and its narrow treatment window (4–5 hrs) limit the number of stroke patients (5 to 10%) receiving such treatment [1]. Numerous neuroprotective agents that have been developed and tested in clinical trials so far have also failed to yield favorable outcomes. Even though neuronal repair therapy has become a viable therapeutic option, its progress has been hampered due to the lack of complete understanding of the underlying mechanisms [2].

MicroRNAs (miRNAs) are a novel class of endogenous, small, non-coding RNAs, comprising of approximately 18–24 nucleotides. miRNAs control gene expression by targeting mRNAs for degradation and/or translational repression. Since the discovery of the first miRNA, lin-4 [3], studies on miRNAs have grown exponentially. To date, miRNAs’ roles have been demonstrated in numerous pathophysiological processes, including stroke. The miRNA expression profiling of cerebral ischemia in both brain and blood was first reported by Jeyaseelan et al [4]. Since then, several studies on miRNA expression in stroke using human samples and animal models have been carried out [5], [6], [7], [8]. These reports implicated the potential functions of miRNAs as biomarkers for diagnosis and prognosis as well as therapeutic targets in cerebral ischemia. However, miRNA expression in stroke recovery process is still poorly understood. This study was designed to identify temporal miRNA expression profiles from acute to recovery phase with more emphasis on the latter in order to understand the neuronal repair processes that follow cerebral ischemia.

## Materials and Methods

### Ethics Statement

All animals were handled according to the Council for International Organisation of Medical Sciences on Animal Experimentation (World Health Organisation, Geneva, Switzerland) and the National University of Singapore (IACUC/NUS) guidelines for laboratory animals. The protocol was approved by the Committee on the Ethics of Animal Experiments of the National University of Singapore (Protocol Numbers: 708/04, 081/09 and 025/11). All surgery was performed as in the approved protocols, and all efforts were also made to minimize suffering.

### Middle Cerebral Artery Occlusion (MCAo) and Quantitation of Infarct Volume

Male Wistar rats (280 g–320 g) were obtained from the Laboratory Animal Centre (National University of Singapore, Singapore) and maintained on an *adlibitum* intake of standard laboratory chow and drinking water. All animals were handled according to the Council for International Organisation of Medical Sciences on Animal Experimentation (World Health Organisation, Geneva, Switzerland) and the National University of Singapore (IACUC/NUS) guidelines for laboratory animals.

Minimum number of animals (n = 6) were used for each category. MCAo was induced via injection of an embolus into the middle cerebral artery [9]. The ipsilateral cerebral blood flow (CBF) was measured by Laser Doppler Flowmetry (OxyFlo, Oxford-Optronix, Oxford, UK). The eMCAo model was considered successful (inclusion) only when cerebral blood flow dropped equal or more than 80% of baseline during occlusion. Furthermore, this blood flow rate was maintained for at least one hour (except for 0 hr time point). The MCA occluded rats were observed closely for the first 1 hour (monitor the CBF using Laser Doppler) and subsequently hourly for another 4–6 hour and thereafter twice a day up to 7 days.

Animals were sacrificed at 0 hrs, 3 hrs, 6 hrs, 12 hrs, 24 hrs, 48 hrs, 72 hrs, 120 hrs and 168 hrs following MCAo and the brain samples were sectioned and subjected to infarct volume quantitation as described previously [10]. Infarct volume was measured by the indirect method formulation described by Swansson et al [11] and Lin et al [12]. This method measures the infarct volumes that eliminate the contribution of edema and swelling. The indirect infarct volume calculation method utilizes the volume of non-infarct area.

Neurological examinations were performed at every time point before euthanasia at 3 hrs, 6 hrs, 12 hrs, 24 hrs, 48 hrs, 72 hrs, 120 hrs and 168 hrs post-operation. The neurological findings [13] were scored with some modifications on a 5-point scale: 0-no neurological deficit; 1- left Horner’s syndrome; 2-failure to extend right forepaw fully; 3-turning to right; and 4-circling to right. We used the following exclusion criteria for our study (a) rCBF drop to <80% of baseline and lasting <1 hour; (b) Neurological score that remain <4 at 3 hrs & 6 hrs and <3 from 12 hrs to 168 hrs; (c) Rats that die during the study period (mortality rate remained low at ∼2% in this study); (d) Rats that show haemorrhagic transformation (∼ 2% in this study).

### RNA Extraction and Micro Arrays

Total RNA (+ miRNA) was extracted from brain tissues by a single-step method using Trizol reagent (Invitrogen, Life Technologies, USA) according to manufacturers’ protocol.

The oligonucleotide (DNA) microarray was performed according to manufacturer’s protocol (Illumina, SanDiego, USA) using 400 ng of total RNA. Data were analysed with the BeadStudio software. Differentially expressed genes were selected based on *p value <0.05*, differential score >20, average signal intensity >100.

miRNA array was performed [14] using total RNA (500 ng) which was 3′-end-labelled with Hy3 dye using the miRCURY LNA™ Power Labeling Kit (Exiqon, Denmark) and hybridized for 16–18 hrs on miRCURY LNA™ Arrays, using MAUI® hybridization system according to manufacturer’s protocol (Exiqon, Denmark). The microarray chips were then washed and scanned using InnoScan700, microarray scanner (Innopsys, Carbonne, France) and analysed on Mapix® Ver4.5 software.

The data discussed in this publication have been deposited in NCBI’s Gene Expression Omnibus [15] and are accessible through GEO Series accession number GSE46269 (http://www.ncbi.nlm.nih.gov/geo/query/acc.cgi?acc=GSE46269).

### Pathway Analyses for miRNAs and mRNAs

Significantly expressed miRNAs (*p<0.05*, fold change>+2 or<−2) were used for pathway analysis for both acute (0 hrs, 3 hrs, 6 hrs, 12 hrs, 24 hrs) and recovery phase (48 hrs, 72 hrs, 120 hrs, 168 hrs). Partek® Genomics Suite™ 6.6 (Partek Inc, USA) and MicroCosm targets version 5 database were used to determine the predicted target genes in the respective pathways. The selected pathways were plotted according to the enrichment score performed by chi-square for each time point.

### miRNA Validation and Measurement of Corresponding mRNAs

Reverse transcription and real-time quantitative PCR (qRT-PCR) were carried out for miRNA and mRNA validation [4]. For miRNA detection reverse transcription followed by stem-loop qRT-PCR reactions were performed according to manufacturer’s protocols using miRNA specific primers from Applied Biosystems (USA).

### Primary Cortical Neuronal Cultures

Primary cultures of cortical neurons were established from E15 Swiss albino mouse brains according to Hirai et al [16] with slight modifications. The cortices were dissected from E15 mouse embryos and washed with Hanks’ balanced salt solution (HBSS, 14025-092, Gibco, Invitrogen, USA). The cortical slices were dissociated with 0.05% (w/v) trypsin in HBSS without Ca^2+^/Mg^2+^ (14175-095, Gibco, Invitrogen, USA) for 30 min at 37°C and neutralized with 1 mg/ml trypsin inhibitor (T6522, Sigma, USA). Single cells were obtained by gentle trituration in Neurobasal medium (21103-049, Gibco, Invitrogen, USA) supplemented with B27 (17504-044, Invitrogen, USA), L-glutamine and Penicillin-streptomycin (Gibco, Invitrogen, USA). The cells were counted by trypan blue exclusion and seeded on to poly-d-lysine coated 24 well plates at a density of 120,000 cells/cm^2^. Cultures were maintained at 37°C with 5% CO_2_ in a tissue culture incubator. The purity level of each culture was evaluated using immunofluorescent techniques.

### Oxygen-glucose Deprivation

Primary neuronal cultures from day 6 were subjected to oxygen-glucose deprivation (OGD) as previously described [17]. Glucose-free Earle’s balanced salt solution (EBSS) was saturated with a mixture gas of 5%CO_2_, 95%N_2_, in a ProOx *in vitro* chamber (BioSpherix, USA) at 37°C overnight, with O_2_ maintained at 0.1%. Day 6 neuronal cultures were washed twice with this medium and incubated for 4 hr in the chamber. OGD was terminated by replacing the glucose-free EBSS with reperfusion medium (Neurobasal medium with L-glutamine and Penicillin-streptomycin, without B27 supplement). Control cultures were treated identically, but without exposure to OGD conditions. During reperfusion, the cells were maintained in a regular 5% CO_2_ incubator for 24 hrs.

### Transfection of miRNAs in Primary Neuronal Cells

Transfection procedures of miRNAs were adapted from Sepramaniam et al [18]. Inhibitor (anti) or miR-206 mimic at 30 nM each (final) in 50 µl of Opti-MEM was complexed with 1 µl of NeoFx in 50 µl of Opti-MEM (Ambion, Inc, USA). Primary neuronal cultures that were subjected to OGD were transfected with these complexes and reperfusion was carried out as described above.

### Morphologic Assessment of Apoptosis and Cell Viability Using Hoechst/Ethidium Homodimer III Nuclear Staining and Fluorescence Microscopy

Cells subjected to OGD (and/or anti/miR-206 mimic) were stained with Hoechst 33342 and Ethidium Homodimer III (EtHD) dye as per the manufacturer’s protocol (Biotium, USA.). Stained cells were protected from light until visualized by fluorescence microscopy (DMIRB, Leica, Leica Microsystems Inc, Deerfield, IL USA). Images were captured at 40× objectives and cell morphology was determined as follows; (1) Viable cells had blue-stained normal, smooth nuclei. (2) Apoptotic cells had blue-stained nuclei with fragmented/condensed chromatin. A minimum of 3 fields of at least 100 cells per field were counted to determine the percentage of apoptotic cells from the total number of cells. Experiments were performed as triplicates.

### Immunocytochemistry

The purity level of each culture was tested using immunofluorescent techniques. The cells were fixed and labelled with a neuronal marker (anti-MAP2, Abcam, USA) and with FITC-labelled secondary antibodies as described by Sepramaniam et al [18]. Images were viewed and analyzed using LSM710 confocal imaging software (Carl Zeiss MicroImaging Inc, USA).

### Statistical Analysis

Microarray analysis involved multiple sample analysis including background subtraction, t-Test/One-way ANOVA analysis, hierarchical clustering [14]. Normalization was performed using 5S rRNA. *t*-Test was performed between “control” and “test” sample groups and the t values were calculated for each mRNA. *p*-values were computed from the theoretical *t*-distribution. The clustering using hierarchical method was performed with average linkage and Euclidean distance metric. The clustering was generated using TIGR MeV (Multiple Experimental Viewer) software and statistical analysis was performed using Partek® Genomics Suite™ 6.6 (Partek Inc, USA). Statistical evaluations were performed using two-tailed t-tests or in case of multiple comparisons using One-way ANOVA with significance level *p value <0.05*. Pearson correlation was used to test the relationship between samples and indicated as Pearson correlation coefficient (R).

## Results

### Embolic Stroke Models Exhibit Spontaneous Recovery from Stroke

**Figure 1:**
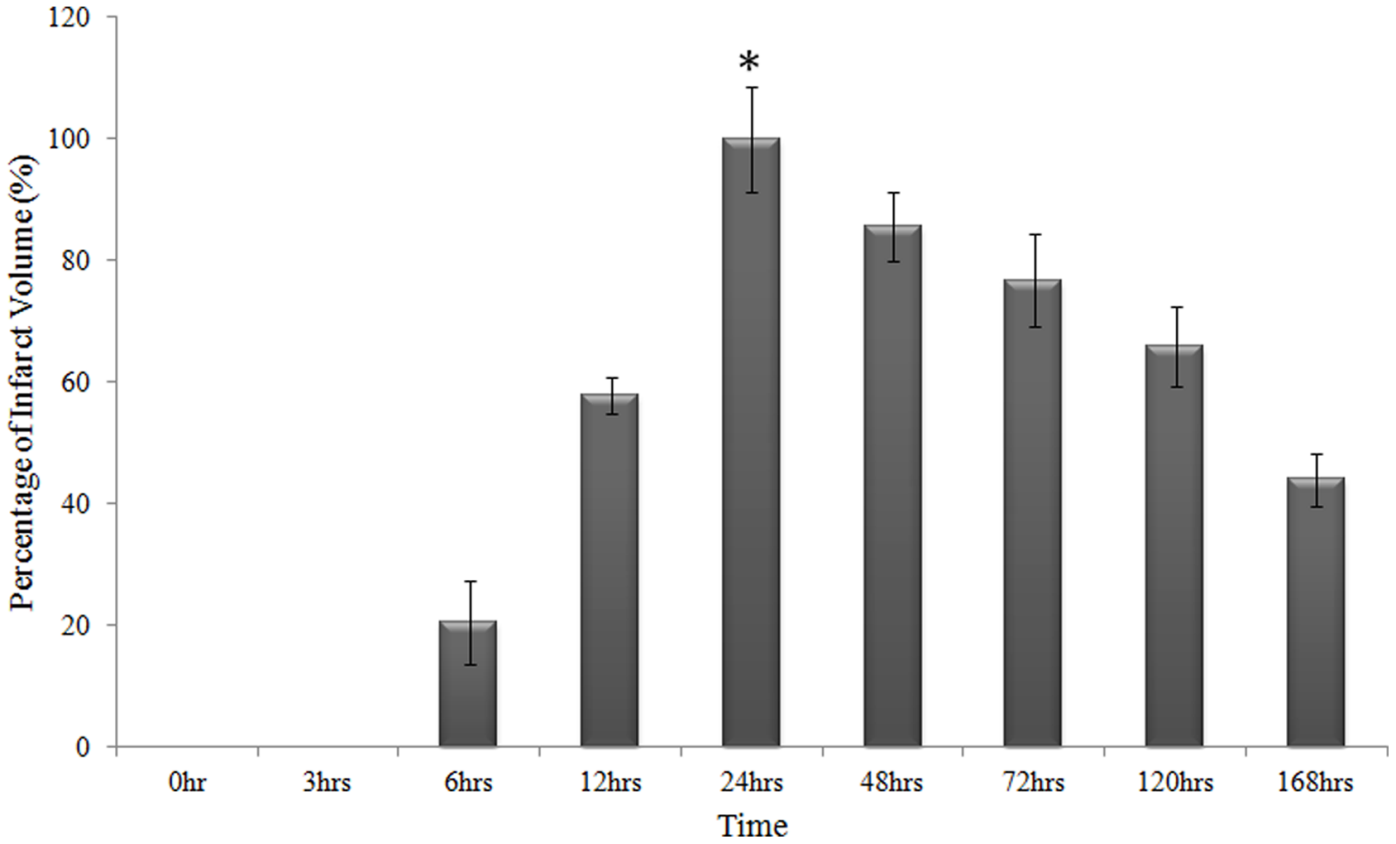
Histogram on percentage of infarct volume at 0 hrs, 3 hrs, 6 hrs, 12 hrs, 24 hrs, 48 hrs, 72 hrs, 120 hrs and 168 hrs after MCAo. Brain slices were stained with TTC prior to the measurement of the infarct size. Infarct volume at 24 hrs post occlusion is significantly the maximum (350±30 mm^3^) than that of other time points (*p<0.05*), thus the infarct volume at 24 hrs is set as 100%.

Embolic rat models (n = 6) subjected to MCAo were sacrificed at different end points such as 0 hr, 3 hrs, 6 hrs, 12 hrs, 24 hrs, 48 hrs, 72 hrs, 120 hrs and 168 hrs. We evaluated the neurological deficit for each animal at different time points (except 0 hrs) before sacrificing and obtaining neurological score of 4 at 3 hrs–6 hrs and ≥3 for 12–168 hrs time points. The brain slices of these rats were stained with TTC and infarct volumes on the ipsilateral region were measured. The infarct volume became visible and measurable only from 6 hrs (20.58±6.86%). The infarct volume peaked at 24 hrs (100±9.52%) and then declined progressively from 48 hrs until 168 hrs (43.99±4.4%) post-occlusion (Figure 1). We included 2 types of control in our analysis. The 0 hr samples (obtained immediately after surgery and introduction of the embolus) were considered as sham operated controls. Another control used for calibration in our gene analysis was the normal rats (without any surgery).

### Changes in miRNA Transcriptome in the Ischemic Brain Upon Embolic Stroke

**Figure 2:**
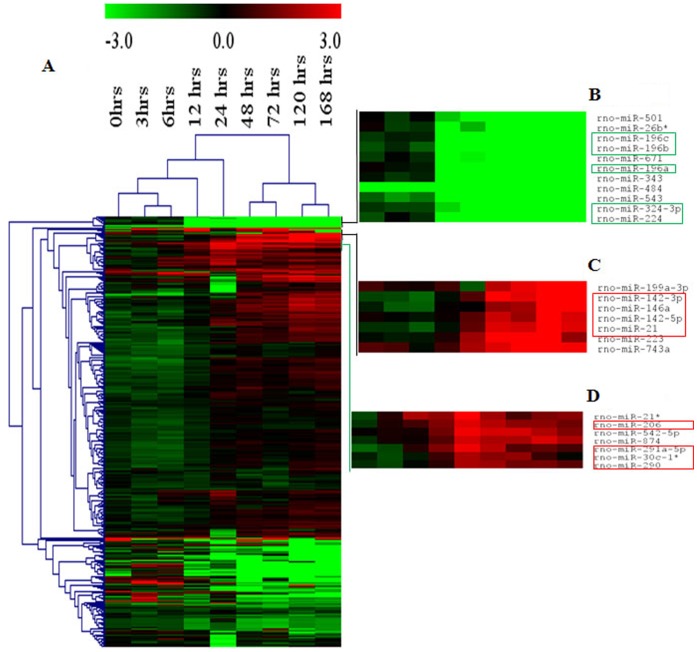
Heat map of the miRNAs that are regulated at different times following MCAo. The signal log ratio values (vs normal rats) were used to construct the hierarchical clustering using the Euclidean distance and average linkage method, carried out on both the samples column and the miRNAs rows. Only significantly different (ANOVA single factor, *p value <0.05*) miRNAs were included for analysis. Green: downregulation; Red: upregulation. (**A**) The expression of 334 miRNAs that are statistically significant are represented in the heat map; (**B**) Cluster of miRNAs that show a decrease in expression during the progression of cerebral ischemia (rno-miR-196a, -196b, -196c, -224, -324-3p); (**C**) Cluster of miRNAs that show an increase in expression during the progression of cerebral ischemia (rno-miR-21, -142-3p/5p, -146a); (**D**) Cluster of miRNAs that show an expression which positively correlated with infarct volume during the progression of cerebral ischemia (rno-miR-206, -290, -291a-5p and -30c-1*).

A total of 346 miRNAs showed differential expression during cerebral ischemia. 12 microRNAs (rno-miR-7b, -138, -143, -181a*, -301a, -328, -330, -336, -376a, 376b-3p, -412, -539) did not fall in the statistically significant (1-way ANOVA *p value <0.05*) category at all time points examined. Hence, the expression pattern of the remaining 334 miRNAs is depicted in the heatmap (Figure 2A).

A large number of miRNAs were downregulated during the initial stages of MCAo. However from 24 hrs post-occlusion, most of these miRNAs demonstrated a reverse trend (upregulation) which continued to increase until 168 hrs (Figure 2A). From these data, three groups of miRNAs exhibiting unique expression patterns were identified (Table S1). The first two groups showed opposing expression patterns. Rno-miR-1*, -10a-5p, -10b, -133a/b, -18a, -196a/b/c, -20b-3p, -224, -26b*, -296*, -297, -301b, -324-3p, -343, -380, -421, -448, -449a, -484, -501, -532-3p, -543, -671 and -96 displayed decreasing expression from 0 hrs to 168 hrs whereas rno-miR-142-3p, -142-5p, -146a, -15b, -17, -181d, -196a*, -199a-3p, -19a/b, -20a, -21, -223, -25, -27a/b, -298, -338, -339-5p, -363*, -374, -382*, -423, -425, -743a and -760-5p displayed increasing expression through all time points. A Pearson Correlation test was also performed by comparing the miRNA expression values against each time point. From the first group, miR-196a/b/c, -224 and -324-3p were found to demonstrate significant downregulation in recovery phase (*p value <0.05*) with R values 0.92, 0.93, 0.92, 0.90 and 0.90 respectively (Figure 2B **)**. Within the second group rno-miR-142-3p, -142-5p, -146a and -21 showed a significant upregulation (*p value <0.05*) in recovery compared to acute phase (Figure 2C **)** and the R values were 0.83, 0.89, 0.86 and 0.92 respectively. Strong correlation values suggest that these clusters of miRNAs could serve as potential biomarkers for diagnosis and prognosis in cerebral ischemia. The third group of miRNAs exhibited an expression profile that reflected the progression of infarct volume. rno-miR-206, -21*, -290, -291a-5p, -300-5p, -30c-1*, -503, -542-5p, -874 and -877 increased in expression from 0 hrs to 24 hrs and gradually decreased from 48 hrs to 168 hrs. Among them, expression of rno-miR-206, -290, -291-5p and -30c-1* (Figure 2D) correlated well with the infarct volumes with R values of 0.96, 0.95, 0.97 and 0.95 respectively.

### Pathways Identified at each Time Point Post-occlusion in Ischemic Stroke

**Figure 3:**
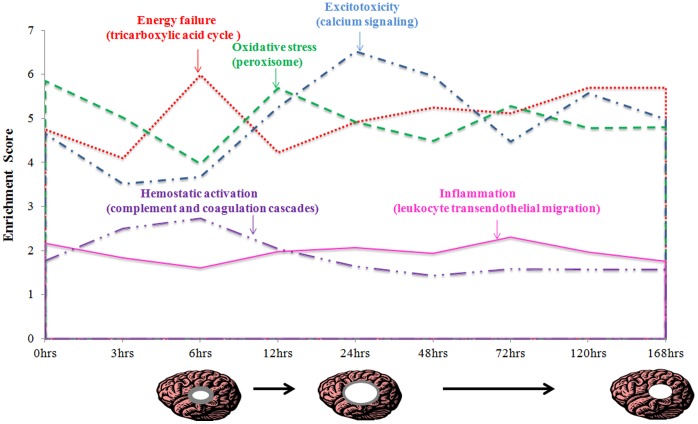
Graph representing the main pathological mechanisms occurring during cerebral ischemia. Infarct area (represented by white circle) becomes visible from about 6 hrs, reaches maximum at 24 hrs and decreases during recovery. The penumbra region which represents the on-going ischemic injury leading to the infarct expansion up to 24 hrs is shown as grey area.

The significantly expressed miRNAs (*p value <0.05*, Signal Log Ratio (SLR)>+1 or<−1) at each time-point were selected for pathway analysis. Using closely related pathways and the enrichment scores obtained from gene dysregulation the progression of the pathological processes in ischemic stroke were followed (Figure 3). Tricarboxylic acid (TCA) cycle pathway was selected to represent energy failure and hypermetabolism, calcium signaling indicated excitotoxicity, peroxisome pathway indicated oxidative stress, leukocyte trans-endothelial migration pathway represented inflammation and complement coagulation cascades reflected processes that occur during haemostatic activation. A sharp increase in the dysregulation of TCA cycle at 6 hrs after the onset of ischemia was observed, suggesting major energy failure. This corresponds to the time point when the infarct volume becomes visible and measurable. Extensive changes in oxidative stress and excitotoxicity followed subsequently and this was probably triggered by the energy failure. Profile of the calcium signaling pathway suggested excitotoxicity peaked at 24 hrs and this correlated with the maximum infarct volume.

### Pathways Involved in the Progression of Cerebral Ischemia

**Figure 4:**
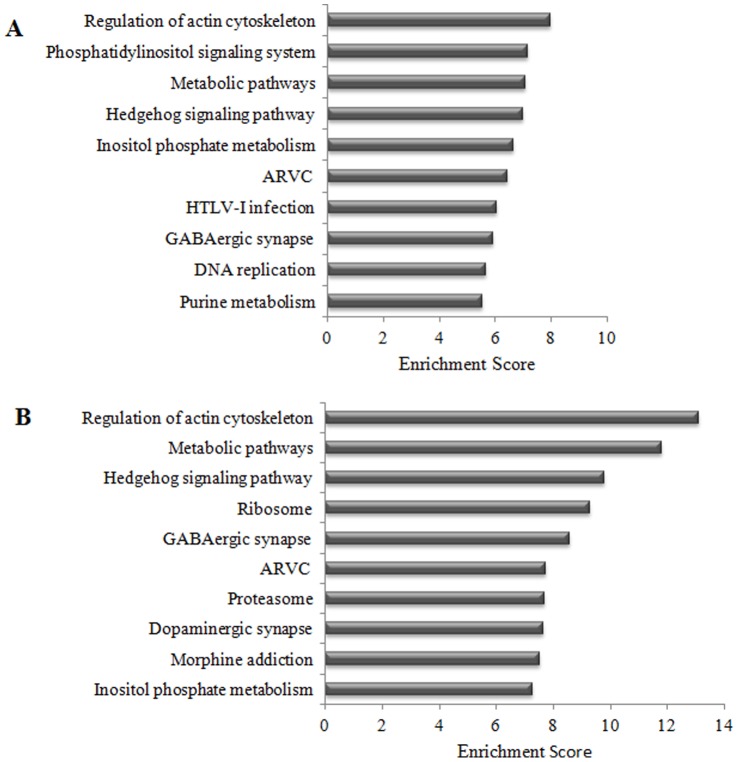
Pathway analysis of miRNAs that were regulated in early time points and late time points after MCAo. Top ten pathways with minimum Enrichment Score of 5.5 were selected. (**A**) Top ten pathways for miRNAs in earlier time points of MCAo model; (**B**) Top ten pathways for miRNAs in later time points of MCAo. ARVC- Arrhythmogenic right ventricular cardiomyopathy.

We next chose to study the differences between the acute and recovery phases. The top ten pathways of acute phase (0 hrs to 24 hrs) for miRNAs examined in this study included the regulation of actin cytoskeleton, phosphatidylinositol signaling system, metabolic pathways, hedgehog signaling pathway, inositol phosphate metabolism, arrhythmogenic right ventricular cardiomyopathy (ARVC), HTLV-1 infection, GABAergic synapse, DNA replication and purine metabolism (Figure 4A). The top ten pathways of recovery phase (48 hrs to 168 hrs) for miRNAs comprised of regulation of actin cytoskeleton, metabolic pathways, hedgehog signaling pathway, ribosome, GABAergic synapse, ARVC, proteasome, dopaminergic synapse, morphine addiction and inositol phosphate metabolism (Figure 4B). Among these, phosphatidylinositol signaling system, HTLV-I infection, DNA replication and purine metabolism were only displayed in acute phase, whereas ribosome, proteasome, dopaminergic synapse, and morphine addiction were only reflected in recovery phase. Three pathways that were common for both acute and recovery phase included regulation of actin cytoskeleton, metabolic pathways, hedgehog signaling pathway.

Eight (GABAergic synapse, ribosome, morphine addiction, regulation of actin cytoskeleton, proteasome, dopaminergic synapse, metabolic pathways and hedgehog signaling pathway) out of the ten pathways shown to be dysregulated by mRNA data were also observed in the analysis of the miRNA data. The remaining two pathways for mRNA expression analysis, neuroactive ligand-receptor interaction and DNA replication were ranked eleventh and fourteenth respectively in miRNA data analysis. These findings suggest a high correlation between both our miRNA and mRNA array data.

### miRNA and mRNA Real-time PCR Validation

**Figure 5:**
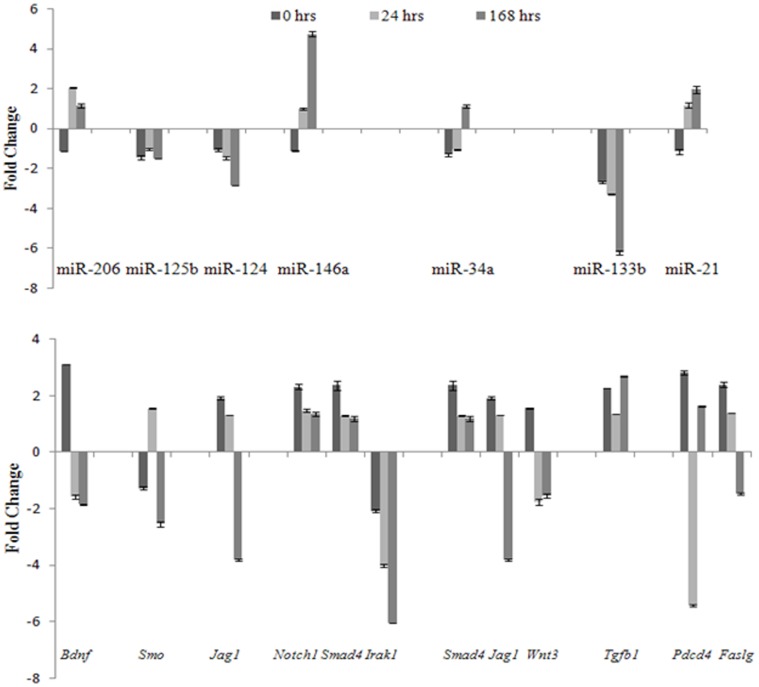
Comparison of miRNA expression with its corresponding mRNA targets measured by quantitative RT-PCR. Data presented as mean ± SEM (n = 3). Target relationship: miR-206/*Bdnf*; miR-125b-5p/*Smo*; miR-124/*Jag1*; miR-146a/*Notch1, Smad4* and *Irak1*; miR-34a/*Smad4, Jag1* and *Wnt3*; miR-133b/*Tgfb1*; miR-21/*Pdcd4* and *Faslg*.

Consistent results were obtained upon validation of mRNA and miRNA array data by qRT-PCR (Figure 5 and Table S2). miR-206 expression peaked at 24 hrs and gradually decreased until 168 hrs while its target, *Bdnf* exhibited an opposing trend. miR-125b-5p showed an opposite profile to *Smo* only at 24 hrs. Brain specific miR-124 was downregulated throughout all the observed time points but its target *Jag1* exhibited upregulation at 0 hrs and 24 hrs. miR-146a consistently increased from 0 hrs to 168 hrs whereas the expression of its targets *Notch1*, *Smad4* and *Irak1* consistently decreased. Similarly, miR-34a expression at 0 hrs, 24 hrs and 168 hrs negatively correlated to its targets, *Smad4, Jag1* and *Wnt3*. miR-133b was found to be downregulated at all time points, while *Tgfb1* remained upregulated. miR-21 demonstrated reverse expression to *Pdcd4* at 0 hrs and 24 hrs while *Faslg* exhibited negative correlation at 0 hrs, 24 hrs and168 hrs.

### miR-206 Expression and Infarct Volume

**Figure 6:**
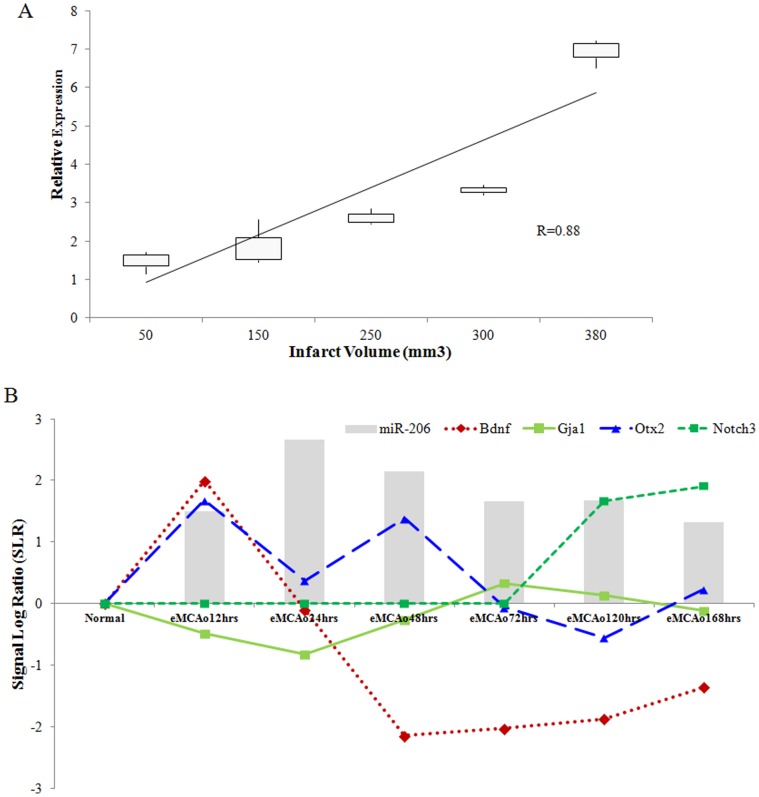
miR-206 correlated with increased cell death in vivo. (**A**) MiR-206 expression as validated by qRT-PCR in an independent cohort of eMCAo models with varying infarct sizes. (**B**) Expression of miR-206 and its target mRNA (*Gja1, Notch3, Otx2 and Bdnf*) in rat brain subjected to MCAo. Inverse correlation was observed between miR-206 and *Bdnf*, *Notch3, Otx2* and *Gja1*.

We measured the miR-206 expression in eMCAo brain samples that were randomly selected at different time points from an independent cohort with varying infarct sizes (averaging from 50 mm^3^ to 380 mm^3^) by quantitative PCR. We observed a positive linear relationship between miR-206 expression and infarct sizes with a Pearson correlation value of R = 0.88 (Figure 6A). We could also see correlations with the expression of corresponding mRNAs that are known to be regulated by miR-206 (Figure 6B).

### miR-206 Expression in Primary Neuronal Cells Upon OGD

**Figure 7:**
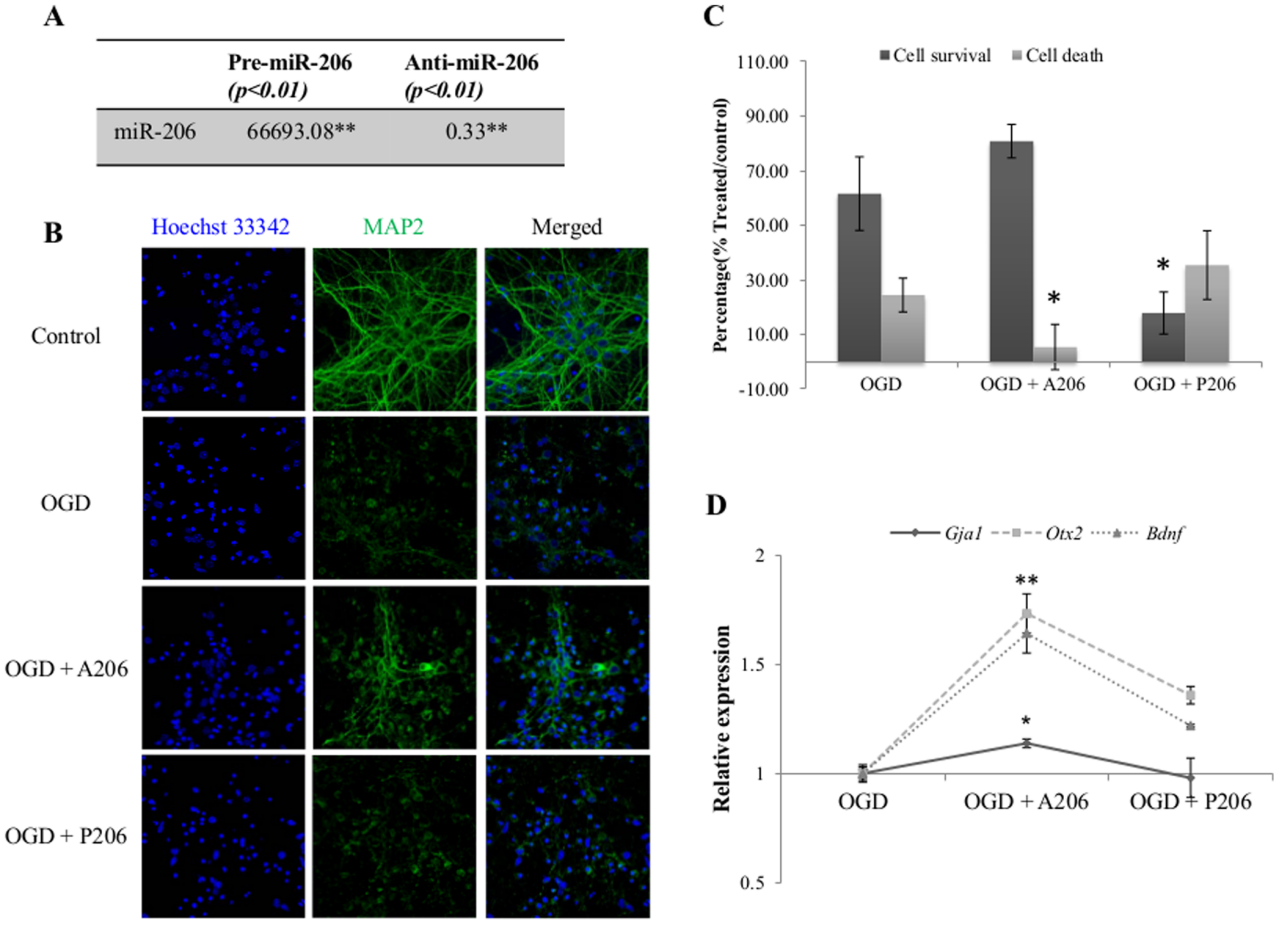
Cell viability and corresponding gene expression in primary neuronal culture transfected with anti-miR-206 or miR-206 mimic during reperfusion following OGD. (A) miR-206 expression is significantly increased in the presence of miR-206 mimic (*p<0.01*) and significantly decreased when cells were transfected with anti-miR-206 (*p<0.01*) during reperfusion. Expression value was calculated as relative expression compared to vehicle transfected controls. (B) Neurons subjected to OGD displayed increased apoptosis and degenerated neurites. Cells transfected with anti-miR-206 after OGD showed increased cell survival and maintenance of neurites as compared to cells subjected to OGD. Cells transfected with pre-miR-206 displayed increased cell death and degeneration of neurites as compared to cells subjected to OGD only. (C) Cells transfected with anti-miR-206 (A206) demonstrated significantly reduced cell death compared to cells subjected to OGD and transfected with vehicle (*p<0.05*), whereas cells transfected with miR-206 mimic (P206) showed significantly increased cell death compared to cells subjected to OGD transfected with vehicle (*p<0.05*). (D) Expression of *Gja1, Otx2* and *Bdnf* showed negative correlation to miR-206 expression. Expression of *Gja1* (*p<0.05*), *Otx2* (*p<0.01*) and *Bdnf* (*p<0.01*) significantly increased in neuronal cells that were transfected with anti-miR-206 during reperfusion period. Relative expression (2^−ΔΔCt^) was calculated based on vehicle transfected controls as calibrator and GAPDH as endogenous control. A206 denotes anti-miR-206; P206 indicates pre-miR-206.

We have measured miR-206 expression in primary neuronal cells during OGD and found miR-206 expression increased significantly at 4 hrs (relative expression value 1.47±0.09, *p<0.05*). Therefore, we selected the 4 hrs OGD time point in our study. We found that miR-206 expression increased significantly (*p<0.01*) when 30 nM miR-206 mimic was added to primary neuronal culture during OGD reperfusion. The miR-206 expression decreased significantly (*p<0.01*) when anti-miR-206 was added (Figure 7A) during the reperfusion incubation period. We also observed that anti-miR-206 could reduce neuronal cell death significantly (Figure 7B, 7C) along with significant increase in *Gja1*, *Otx2* and *Bdnf* expression (Figure 7D).

## Discussion

### Spontaneous Recovery in Ischemic Stroke

This study for the first time reports the changes in miRNA expression both in acute and recovery phases in embolic rat stroke models. Clinically, stroke patients display improvement over several days. Maximum arm motor function can be achieved in patients (95%) within 9 weeks of stroke onset [19] whereas the final level of language function can be achieved (95%) by 6 weeks post-stroke [20]. Animal studies have also provided evidence of spontaneous recovery from stroke [21]–[24]. Furthermore, Lu et al [25] have also shown that infarct volume reaches a maximum at 24 hr reperfusion and reduces gradually thereafter. The team had observed a temporal gene expression profile for these time points and correlated them with a decrease in infarct volume during 3 and 7 days reperfusion as well as to neural repair mechanisms including glial proliferation around/into the infarct region. In our study, we have used 2 control groups to explain our observations. In our study, we have used 0 hr and normal groups for our gene expression analysis. While the 0 hr samples were considered to be equivalent to sham (surgery+embolus), normal (non surgical) group was used in calibrating the gene expression changes upon injury as well as during the recovery process. From this study, we have identified potential miRNAs that could possibly be involved in the regulation of these recovery processes from the onset of ischemic stroke.

### MiRNAs in Acute and Recovery Phases of Ischemic Stroke

Most of the miRNAs were found to be downregulated in the acute phase (0 h to 24 hrs) of ischemia. They became upregulated during the recovery phase (48 hrs to 168 hrs). We found miR-21 and miR-146a to be highly expressed in recovery phase compared to the acute phase of stroke and hence might be useful in predicting the recovery process (Figure 5, Table S1). miR-21 is known to prevent apoptosis by targeting *Faslg* [26]. miR-146a was reported to elicit neuroprotective effect by targeting *Irak1* [27]. In contrast, miR-196a/b/c, miR-224 and miR-324-3p were found to be downregulated, hence their low expression may also be indicative of recovery. Furthermore, miR-206, miR-290, miR-291-5p and miR-30c-1* expression was found to be positively correlating with the infarct volume. In this study we could demonstrate the correlation between miR-206 and infarct volume. Previously, our group [4] and Li et al [28] reported that upregulated miR-290 could inhibit *Vsnl*1 and subsequently reduce excitotoxicity and infarct volume.

### Pathway Analysis of Dysregulated miRNAs

Pathway analysis indicated energy failure, excitotoxicity, oxidative stress, inflammation and haemostatic activation (Figure 3). Energy failure peaking at 6 hrs could trigger oxidative stress and subsequently enhance excitotoxicity at 12 and 24 hrs. Moreover, we identified phosphatidylinositol signalling system, HTLV-I infection, DNA replication and purine metabolism to be unique to acute phase (0 hrs to 24 hrs), while ribosome, proteasome, dopaminergic synapse, and morphine addiction to be crucial for recovery processes (48 hrs to 168 hrs). Phosphatidylinositol signaling could activate excitotoxicity and other processes including DNA damage and repair [29]. This followed by ribosome and proteasome activity in recovery phase indicated that DNA repair mechanisms are in operation for recovery [29]. Furthermore, the late phase active dopaminergic synapses, GABA synapses and morphine addiction pathway may reflect the recovery in neuronal transmitter [30].

### miRNAs in Neuronal Repair after Ischemic Stroke

Cerebral ischemia can also activate neuronal stem and precursor cells (NSC and NPC), to migrate to the injured area. These cells contribute to angiogenesis, neurogenesis and synaptogenesis [31]. Hedgehog, Notch, Wnt and TGF-β signaling pathways are all highly conserved pathways [32]–[35] found to be responsible for fostering NSC and NPC proliferation, migration and differentiation to promote neuronal repair following ischemic stroke [36]–[41]. In our study, the genes of the above-mentioned pathways were found to be upregulated, which indicated the participation of these pathways in post ischemic recovery. Hedgehog signalling pathway was ranked as the top four pathways in both early phase and late phase while TGF-β, Wnt and Notch signaling pathways ranked higher in the late phase of ischemic stroke with statistically significant enrichment *p values* (Table 1).

**Table 1:** Four selected pathways and their ranks in early and late time points.

Signaling Pathway	Early time point rank (%)	Enrichment *p-value*	Late time point rank (%)	Enrichment *p-value*
**Hedgehog**	1.6%	*0.0009*	1.1%	*5.61E-05*
**TGF-β**	35.0%	*0.14*	15.0%	*0.01*
**Wnt**	20.0%	*0.06*	16.0%	*0.01*
**Notch**	25.0%	*0.08*	22.0%	*0.03*

Significantly expressed miRNAs (*p value <0.05*, Fold change>+2 or<−2) were selected for pathway analysis for both acute phase (0 hrs to 24 hrs) and recovery phase (48 hrs to 168 hrs) processes. The ranks of Hedgehog, TGF-β, Wnt and Notch signaling pathways are presented as a percentage (Total pathway number as 100%). Enrichment *p value* (Partek Pathway Analysis) was used to indicate pathway’s significance (*p value <0.05*).

**Figure 8:**
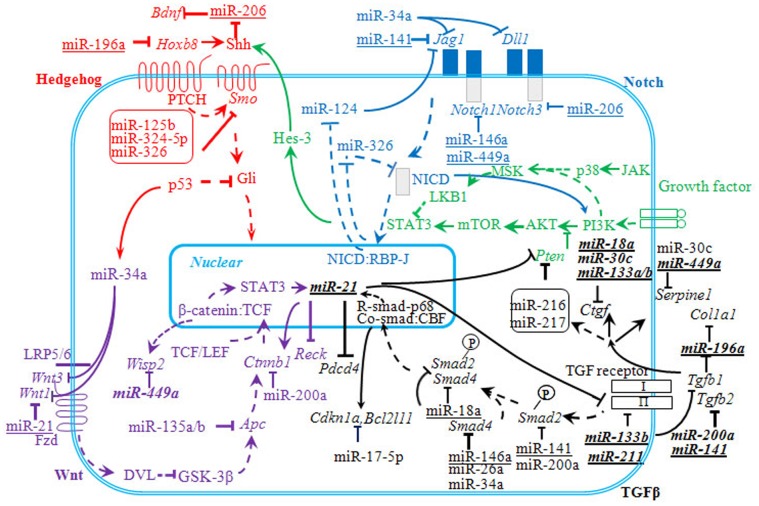
Hedgehog, Notch, Wnt and TGF-β signaling pathways showing mRNAs and the miRNAs involved. Fibrosis-related miRNAs are shown in bold italic fonts. The miRNAs selected from this study are underlined. All targets for miRNAs were selected from published and validated data. *mTOR* mammalian target of rapamycin; *PI3K* -Phosphatidylinositol 3-kinases; *STAT3* -signal transducer and activator of transcription 3; *Pdcd4* -programmed cell death 4; *Reck* -reversion-inducing-cysteine-rich protein with kazal motifs; *Dll1* -delta-like 1; *Jag1* jagged 1; *WISP2* -WNT1-inducible signaling pathway protein 2; *Shh -*sonic hedgehog; *Smo* Smoothened; *Bdnf -*brain derived neurotrophic factor; *Hoxb8 -*homeobox B8; NICD -Notch internal cellular domain; *Hes3 -*hairy and enhancer of split 3; *Fzd -*Frizzled; *Dvl* -dishevelled; *Gsk3b* -glycogen synthase kinase-3beta; *Apc -*adenomatous polyposis coli; *Tgfbr2* -transforming growth factor beta receptor 2; *Ctgf -*connective tissue growth factor.

The function of miRNAs in neuronal repair following cerebral ischemia is still poorly understood. Nevertheless, their ability to regulate the above mentioned pathways have been demonstrated in cancer and cardiovascular diseases [42]–[45]. The miRNA and mRNA interaction in the selected pathways could trace the interaction of these four main pathways (Figure 8). The *Pten* gene was seen to be the common link between these four pathways. Notch signalling is known to activate Hedgehog pathway through PI3K/AKT and mTOR activation [38], which is also induced by TGF-β signalling. *Pten*, the inhibitor of PI3K/AKT, was targeted by miR-216 and miR-217 and these miRNAs were also activated by *Tgfb*1 [46]. miR-21 linked Wnt and TGF-β signalling [47], [48]. Therefore, it is possible that these four pathways could cross-talk with each other to regulate neuronal repair. From our profiling data, we also observe that miRNA families such as miR-206/−133b; miR-200a/−141; miR-34a/−449a; miR-17-5p/−18a may be working together to regulate neuronal repair mechanisms. Evidently, we observed miR-206 expression increases with increase in infarct volume (Figure 6A). Hence, the level of miR-206 expression could be used in determining the progression of recovery or specifically the reduction in infarct volume.

miR-21 that is overexpressed in most tumor types is known to act as an oncogene by targeting many tumor suppressor genes required for proliferation, apoptosis, and invasion [49]. It involves TGF-β/Smad signalling, β-catenin/STAT3 signaling [48], [50] and genes like *PTEN* [51], *Pdcd4* [52], *Faslg* [26], and *RECK* [48]. In our study, miR-21 was found to be significantly upregulated following cerebral ischemia, indicating that it may play a similar role in cerebral ischemia as a NPC regulator.

miR-34a causes cell cycle arrest and apoptosis in cancer stem cells [53] *via* p53. miR-34a was also reported to target Notch signaling pathway to inhibit tumor stem cell invasion by directly binding to *NOTCH1, DLL1* and *JAG*1 [54], [55] as well as Wnt signaling pathway by targeting *WNT1* and *WNT3* genes [53], [56] and TGF-β signaling pathway *via Smad4* [57]. In our study, *Tp53* and miR-34a were upregulated, hence could inhibit Notch, Wnt and TGF-β signaling by targeting *Dll1, Jag1, Wnt1, Wnt3* and *Smad4. Tp53* was also reported to inhibit Hedgehog signalling [58], implicating that p53 and miR-34a may function as negative regulators of NPC proliferation by inhibiting Notch, Wnt, Hedgehog and TGF-β signaling pathway following cerebral ischemia. Furthermore, miR-449a was also reported to bring about cell cycle arrest and apoptosis by a partially p53-independent mechanism in cancer stem cells [59], [60]. In our study miR-449a was downregulated from 12 hrs to 168 hrs, suggesting that it may have a different role from that of miR-34a in regulating the recovery processes in cerebral ischemia.

### Fibrosis in Cerebral Ischemia Recovery

In addition to the above pathways, we have identified miRNAs that could be involved in fibrosis that follows cerebral ischemia. Fibrosis protects the host from an injurious event by deposition of matrix, disruption of the normal tissue architecture, and parenchymal destruction [61]. Astrocytes in the central nervous system become reactive in response to tissue damage leading to the formation of the glial scar. The damaged tissue scar formation in the injured brain is beneficial to limit the extension of damage. We have found a cluster of miRNAs that are highly downregulated during recovery phases (*p<0.05* compared to acute phase; Figure 2A, Table S1). The search for their validated targets showed that several of them (miR-18a, -133a/b, -141, -196a, -211, -324-3p and -449a) were linked to fibrosis. Except for miR-324-3p [62], all of them have been found to be involved in TGF-β signaling pathway regulation. miR-449a also targeted *WISP2* in Wnt signaling pathway. The validated mRNA targets to miRNAs and their involvement in selected pathways are presented in Figure 8. miR-21, -30c and -200a were related to fibrosis by regulating extracellular matrix accumulation [45], [63], [64]. Guo et al [65] reported TGF-β and Wnt signaling pathways are involved in fibrosis. Doyle et al [66] also demonstrated that TGF-β increased reactive astrogliosis post stroke. In addition, Lu et al [25] proposed that decrease in infarct volume at 3 and 7 days reperfusion following MCAo could be facilitated by the glial proliferation around the infarct region. Therefore these two pathways may function in tandem to contribute to recovery following cerebral ischemia.

### miR-206 and Cerebral Ischemia

miR-206, is a muscle-specific miRNA and is a key regulator of muscle cells proliferation, differentiation, apoptosis, migration and angiogenesis [51], [67]–[70]. Furthermore, in HeLa cells, miR-206 was reported to promote apoptosis through inhibition of *Notch3* expression, thus resulting in inhibition of tumor cell migration [71]. In myocardial infarction, the upregulated miR-206 induces cell apoptosis *via* insulin-like growth factor 1 (*Igf1*) [72]. Moreover, the nes-igf1r^(−/wt)^ mice showed increased cerebral infarct volume and neuronal damage upon hypoxia injury [73]. Although miR-206 is poorly expressed (undetectable level) in normal brains, a high level expression of miR-206 has been observed in AD patients and the AD transgenic mouse models. Overexpression of miR-206 in cultured primary mouse hippocampal neurons resulted in a decrease in the dendritic spines density. Correspondingly, low densities of dendritic spines were also observed in the primary hippocampal neurons from the AD model [74]. The authors showed that miR-206 participates in the pathogenesis of AD by suppressing *Bdnf* expression. The authors further demonstrated that intranasal delivery of miR-206 antagomiR improved memory function and increased *Bdnf* levels, while the intracerebral injection of the antagomiR enhanced the memory function, increased synaptic density and neurogenesis in AD (Tg2576) mice. We have observed that the expression of miR-206 to be upregulated in the brain of rats subjected to MCAo. The expression of miR-206 positively correlated with infarct volume expansion in the eMCAo model. Reduction in both miR-206 and infarct volume was observed when the rats subjected to MCAo were administered with MK-801 and PLA_2,_ to reverse the ischemic brain injury [10]. Gibson et al [75] have also reported that estrogen reduces infarct volume in a dose-dependent manner. Subsequently, Leivonen et al [76] have demonstrated that miR-206 could target estrogen receptor-α and repress estrogen receptor-α responsive gene. In another study, overexpression of miR-206 was found to inhibit the neural cell viability [77]. miR-206 induced abnormal development of neural cells was considered to be partly mediated via inhibition of orthodenticle homeobox 2 (*Otx2*) mRNA transcript and translation. *Otx2* plays an important role in neurodevelopment during embryogenesis [78], [79]. During the abnormal development of nerve cells, miR-206 affects cell viability and apoptosis, mainly through regulating the expression of *Otx2* [80]. Another target of miR-206 is the *Cx43* (gap junction protein, alpha 1; *Gja*1), a primary component of gap junction proteins, the intercellular channels in astrocytes. Cx43 is also widely expressed in mouse neural crest cell lineages and has been shown to be vital in neural crest development [81], [82]. *Cx43* has been reported to be crucial for the communication between astrocytes and neurons and normal astrocytic gap junction intercellular communication protected neuron from oxidative stress and glutamate cytotoxicity [83], [84]. During cerebral ischemia, *Cx43^+/−^* mice exhibited a significantly larger infarct volume and greater apoptotic neurons than their wild-type (*Cx43^+/+^*) counterparts [85], [86]. We have also observed that increase in miR-206 expression reflected an increase in cerebral infarct volume while correlating to the downregulation of the *Gja1* mRNA (Figure 6B). Therefore, it is possible that miR-206 promotes acute cerebral cell death by targeting *Cx43* co-operatively with *Bdnf, Notch3 and Otx2* (Figure 6B).

This observation is further supported in our primary neuronal culture study (Figure 7). Neuronal cell death following OGD is significantly reduced by anti-miR-206 that was added during reperfusion period. Correspondingly, *Gja1*, *Otx2* and *Bdnf* expression was also significantly increased. In contrast, increased cell death was observed when miR-206 mimic was added during the reperfusion.

### Conclusions

Thus far, miRNA expression studies in ischemic stroke in both human and animal model have established their importance in acute phase of ischemic stroke. Using expression profiling data and validating corresponding miRNAs to mRNA targets, we have proposed that Hedgehog, Notch, Wnt, mTOR and TGF-β pathways may be involved in bringing about the recovery process in the post ischemic phase of stroke. These biological processes are further assisted by fibrosis that limits the expansion of injury. Further mechanistic studies are required to confirm these findings. Our study also highlights that the miRNAs such as miR-206 could be involved in recovery processes to bring about a favorable outcome in cerebral ischemia.

## Supporting Information

Table S1 **MicroRNA profiling data for selected three categories miRNAs.**
(DOC)

Table S2 **Signal log ratio (SLR) values for selected mRNAs and miRNAs.**
(DOC)
