# Supplementary material for: microRNAs Involved in Regulating Spontaneous Recovery in Embolic Stroke Model
Source: PLoS One. 2013 Jun 18;8(6):e66393. doi: 10.1371/journal.pone.0066393 (PMC3688919; doi:10.1371/journal.pone.0066393)
Supplement: Table S1 — MicroRNA profiling data for selected three categories miRNAs. (DOC) [file pone.0066393.s001.doc]

**Table S1: MicroRNA profiling data for selected three categories miRNAs.** Signal log ratios of miRNAs which were significantly altered across all time points with statistical significance (1-way ANOVA *p value < 0.05*) are listed. These miRNAs showed distinct expression pattern that correlates to either acute phase, recovery phase (**bold font**) or infarct volume (grey box).

| **miRNA** | **SLR values** | | | | | | | | |  |
| --- | --- | --- | --- | --- | --- | --- | --- | --- | --- | --- |
|  | **0hrs** | **3hrs** | **6hrs** | **12hrs** | **24hrs** | **48hrs** | **72hrs** | **120hrs** | **168hrs** | ***p value*** |
| rno-miR-1* | 0.63 | -1.45 | -1.08 | -2.80 | -1.61 | -4.52 | -4.72 | -4.09 | -4.25 | *1.89E-07* |
| rno-miR-10a-5p | 1.10 | 1.73 | 1.35 | 0.62 | -1.30 | -5.37 | 0.43 | -4.94 | -5.09 | *0.001211* |
| rno-miR-10b | -0.03 | 1.78 | -0.69 | -1.91 | -0.51 | -3.54 | -3.73 | -3.10 | -3.26 | *2.09E-07* |
| rno-miR-133a | -0.60 | -1.27 | -0.52 | -0.95 | -4.14 | -1.54 | -1.65 | -6.78 | -2.50 | *7.86E-06* |
| rno-miR-133b | -0.04 | 0.50 | 1.50 | -0.03 | -1.86 | -1.14 | -6.43 | -5.80 | -5.96 | *8.9E-08* |
| rno-miR-141 | -2.22 | 0.56 | -0.42 | -3.25 | -1.53 | -5.61 | -5.80 | -0.15 | -5.33 | *5.45E-16* |
| rno-miR-196a | 0.10 | -0.18 | -0.32 | -4.04 | -3.63 | -7.00 | -7.19 | -6.56 | -6.72 | *5.48E-09* |
| rno-miR-196b | -0.92 | -0.46 | -1.11 | -3.69 | -3.12 | -6.67 | -6.86 | -6.23 | -6.39 | *0.016733* |
| rno-miR-196c | -0.64 | -0.34 | -0.41 | -3.62 | -3.99 | -6.49 | -6.68 | -6.06 | -6.22 | *8.53E-08* |
| rno-miR-18a | -3.15 | 1.01 | -0.76 | -4.15 | -3.48 | -4.45 | -4.64 | -4.02 | -4.17 | *3.81E-05* |
| rno-miR-20b-3p | -2.32 | -0.45 | 0.96 | -3.06 | -1.90 | -6.05 | -6.24 | -5.61 | -5.77 | *1.42E-09* |
| rno-miR-211 | -0.09 | -0.41 | 0.70 | 0.20 | -3.19 | -1.85 | -1.60 | -6.37 | -6.53 | *1.41E-06* |
| rno-miR-224 | -0.93 | -0.06 | -0.51 | -3.60 | -3.16 | -5.50 | -5.69 | -5.06 | -5.22 | *0.000809* |
| rno-miR-26b* | 0.16 | -0.74 | -0.57 | -4.01 | -2.12 | -7.11 | -7.30 | -6.68 | -6.83 | *0.00031* |
| rno-miR-296* | -1.54 | -0.89 | -0.55 | -1.86 | -3.80 | -2.40 | -4.75 | -7.92 | -8.07 | *0.005702* |
| rno-miR-297 | 0.10 | -0.35 | 0.60 | -0.08 | -2.51 | -4.20 | -2.22 | -2.14 | -2.15 | *0.000444* |
| rno-miR-301b | -0.47 | 1.47 | 0.56 | -1.71 | -1.25 | -4.38 | -4.57 | -3.95 | -4.11 | *0.000112* |
| rno-miR-324-3p | -0.60 | -0.79 | -0.80 | -2.54 | -4.29 | -5.81 | -3.63 | -7.83 | -7.99 | *9.74E-07* |
| rno-miR-343 | -0.20 | -0.64 | -0.40 | -3.99 | -3.27 | -7.09 | -7.28 | -6.66 | -6.81 | *0.022446* |
| rno-miR-380 | -0.23 | 1.73 | 0.77 | -1.85 | -0.84 | -4.76 | -4.95 | -4.32 | -4.48 | *2.88E-06* |
| rno-miR-421 | -1.70 | 0.54 | 0.61 | -2.36 | -2.13 | -5.29 | -5.48 | -4.85 | -5.01 | *2.78E-05* |
| rno-miR-448 | 1.15 | 0.35 | 0.57 | 0.75 | 0.18 | -4.94 | -3.58 | -6.46 | -1.67 | *0.01316* |
| rno-miR-449a | -0.02 | -0.54 | 0.48 | -1.43 | -4.22 | -2.35 | -2.70 | -3.24 | -7.48 | *0.00201* |
| rno-miR-484 | -3.04 | -2.98 | -4.41 | -4.71 | -5.12 | -8.89 | -9.08 | -8.46 | -8.61 | *5.5E-14* |
| rno-miR-501 | 0.02 | -0.69 | -0.01 | -2.35 | -3.70 | -7.50 | -7.69 | -7.07 | -7.23 | *9.41E-08* |
| rno-miR-532-3p | -1.58 | -0.80 | -0.38 | -1.08 | -2.15 | -5.96 | -6.15 | -5.53 | -5.69 | *0.000291* |
| rno-miR-543 | -1.14 | -1.82 | -1.41 | -5.09 | -4.70 | -8.41 | -8.60 | -7.98 | -8.14 | *3.05E-07* |
| rno-miR-671 | -0.70 | -0.06 | -0.49 | -3.65 | -2.86 | -6.96 | -7.15 | -6.52 | -6.68 | *5.43E-08* |
| rno-miR-96 | 2.83 | -0.19 | -0.43 | -3.05 | -2.68 | 4.22 | -6.29 | -5.66 | -5.82 | *0.000114* |
| **rno-miR-142-3p** | -0.77 | -0.84 | -1.21 | -0.14 | 0.94 | 3.36 | 2.71 | 4.65 | 4.01 | *2.46E-13* |
| **rno-miR-142-5p** | 0.22 | -0.05 | -0.25 | 0.15 | 1.01 | 2.37 | 1.82 | 3.41 | 2.56 | *6.89E-06* |
| **rno-miR-146a** | -0.33 | -1.04 | -1.11 | 0.09 | 0.00 | 1.71 | 1.93 | 3.52 | 3.15 | *5.47E-12* |
| **rno-miR-15b** | -0.35 | -0.57 | -0.87 | -0.35 | -0.14 | 1.14 | 1.39 | 2.02 | 1.54 | *3.8E-08* |
| **rno-miR-17** | -0.49 | -0.68 | -0.68 | -0.16 | 0.41 | 1.12 | 1.28 | 1.83 | 1.66 | *0.00136* |
| **rno-miR-181d** | -1.27 | -0.23 | -0.90 | -0.10 | 0.47 | 0.39 | 0.82 | 0.80 | 0.99 | *0.003132* |
| **rno-miR-196a*** | -0.17 | -0.23 | 0.17 | 0.40 | -0.23 | 0.35 | 0.77 | 1.04 | 1.14 | *1.14E-06* |
| **rno-miR-199a-3p** | 0.80 | 0.34 | 0.24 | 0.74 | -1.00 | 2.17 | 2.76 | 4.12 | 3.53 | *4.91E-13* |
| **rno-miR-19a** | -0.55 | 0.10 | -0.42 | -0.14 | 0.39 | 1.23 | 1.35 | 2.37 | 1.82 | *2.29E-08* |
| **rno-miR-19b** | -0.69 | -0.16 | -0.59 | -0.16 | 0.23 | 1.23 | 1.33 | 2.34 | 1.98 | *2.45E-09* |
| **rno-miR-20a** | -0.54 | -0.54 | -1.05 | -0.19 | 0.22 | 1.13 | 1.21 | 1.85 | 1.72 | *4.7E-06* |
| **rno-miR-21** | -0.55 | -0.54 | -1.04 | -0.18 | 1.13 | 2.62 | 2.48 | 3.07 | 2.65 | *9.1E-18* |
| **rno-miR-223** | -0.13 | -0.32 | -0.26 | 0.36 | 1.87 | 4.85 | 4.14 | 4.01 | 1.73 | *7.3E-15* |
| **rno-miR-25** | -0.84 | -0.13 | -0.83 | -0.36 | -0.22 | 0.52 | 1.04 | 1.56 | 1.34 | *1.98E-06* |
| **rno-miR-27a** | -0.51 | 0.20 | -0.59 | -0.17 | 0.62 | 0.91 | 1.23 | 2.01 | 1.41 | *5E-06* |
| **rno-miR-27b** | -0.81 | 0.55 | -0.02 | 0.11 | -1.56 | 0.46 | 1.14 | 1.47 | 1.43 | *8.63E-06* |
| **rno-miR-298** | 0.37 | -0.37 | 0.30 | 0.33 | 0.18 | 0.51 | 1.04 | 1.71 | 1.20 | *1.61E-11* |
| **rno-miR-338** | -1.99 | -1.28 | -1.88 | -0.61 | -0.39 | 0.94 | 0.92 | 1.49 | 1.39 | *3.47E-06* |
| **rno-miR-339-5p** | -0.02 | -0.83 | -0.21 | -0.12 | -0.46 | 1.05 | 0.92 | 2.23 | 2.05 | *0.011019* |
| **rno-miR-363*** | -0.93 | 0.51 | 0.25 | 0.01 | -0.61 | 0.13 | 1.25 | 1.87 | 1.46 | *7.63E-05* |
| **rno-miR-374** | -0.93 | -0.34 | -0.85 | -0.38 | 0.05 | 0.48 | 0.68 | 1.54 | 1.45 | *0.000711* |
| **rno-miR-382*** | -0.17 | -0.57 | -0.35 | 0.51 | 1.42 | 1.15 | 1.08 | 1.37 | 1.52 | *0.011576* |
| **rno-miR-423** | -0.53 | -0.76 | -0.37 | 0.48 | 1.41 | 1.23 | 1.26 | 1.68 | 1.41 | *4.15E-05* |
| **rno-miR-425** | -0.30 | -0.39 | -0.53 | 0.05 | 0.70 | 1.07 | 1.20 | 1.90 | 1.72 | *0.000242* |
| **rno-miR-743a** | 0.99 | 0.85 | -0.14 | 0.74 | 2.00 | 2.64 | 3.69 | 3.82 | 2.90 | *0.00036* |
| **rno-miR-760-5p** | 0.99 | -1.24 | -1.09 | 1.01 | 1.42 | 2.16 | 2.11 | 2.09 | 2.13 | *1.1E-07* |
| rno-miR-206 | -0.59 | 0.36 | 0.77 | 1.49 | 2.67 | 2.15 | 1.67 | 1.67 | 1.32 | *3.08E-11* |
| rno-miR-21* | -0.83 | 0.44 | 2.03 | 1.72 | 3.53 | 1.90 | 0.85 | 1.46 | 1.62 | *3.93E-06* |
| rno-miR-290 | -0.53 | -0.81 | -0.11 | 0.48 | 2.00 | 1.87 | 1.33 | 1.39 | 1.05 | *0.001202* |
| rno-miR-291a-5p | -1.16 | -0.79 | 0.45 | 1.48 | 2.83 | 2.19 | 0.96 | 1.15 | 0.82 | *0.000846* |
| rno-miR-300-5p | -0.64 | -0.29 | 1.02 | 0.95 | 2.25 | 0.92 | 0.47 | 0.57 | 0.59 | *0.049824* |
| rno-miR-30c-1* | -0.34 | -0.85 | -0.29 | 0.70 | 2.00 | 1.62 | 1.74 | 0.68 | -0.07 | *1.78E-09* |
| rno-miR-503 | -0.41 | -0.48 | 0.56 | 0.67 | 2.28 | 1.01 | 0.50 | 0.73 | 0.54 | *0.000286* |
| rno-miR-542-5p | 0.09 | 0.29 | 0.24 | 1.32 | 2.86 | 2.53 | 2.54 | 2.07 | 1.52 | *0.008303* |
| rno-miR-874 | -0.52 | 0.25 | 0.15 | 1.00 | 2.49 | 2.03 | 2.01 | 2.57 | 2.04 | *0.000225* |
| rno-miR-877 | -0.43 | -0.24 | 1.32 | 1.31 | 2.13 | 0.50 | 0.61 | 0.93 | 0.71 | *0.000194* |
