# Supplementary material for: microRNAs Involved in Regulating Spontaneous Recovery in Embolic Stroke Model
Source: PLoS One. 2013 Jun 18;8(6):e66393. doi: 10.1371/journal.pone.0066393 (PMC3688919; doi:10.1371/journal.pone.0066393)
Supplement: Table S2 — Signal log ratio (SLR) values for selected mRNAs and miRNAs. (DOC) [file pone.0066393.s002.doc]

**Table S2: Signal log ratio (SLR) values for selected mRNAs and miRNAs.** Validated targets of miRNAs in Hedgehog, Notch, TGF-β and Wnt signaling pathways were selected based on the expression pattern from our study. The SLR values for these *mRNA*s that are targeted by specific miRNAs were extracted from our mRNA and miRNA array data.

| **mRNAs : miRNAs** | **SLR Values** | | | | | | **mRNAs : miRNAs** | **SLR Values** | | | | | |
| --- | --- | --- | --- | --- | --- | --- | --- | --- | --- | --- | --- | --- | --- |
|  | **12hrs** | **24hrs** | **48hrs** | **72hrs** | **120hrs** | **168hrs** |  | **12hrs** | **24hrs** | **48hrs** | **72hrs** | **120hrs** | **168hrs** |
| *Apc* | -0.11 | -1.14 | -1.20 | -1.68 | -2.19 | -0.22 | *Serpine1* | 3.76 | 5.16 | 2.79 | 3.25 | 1.62 | 0.75 |
| rno-miR-135a | -0.52 | -0.20 | 0.06 | -0.12 | 0.22 | 0.21 | rno-miR-30c | -0.44 | 0.07 | 0.30 | -0.10 | -0.03 | 0.28 |
| rno-miR-135b | -0.38 | -0.11 | 0.11 | 0.04 | 0.22 | 0.11 | rno-miR-449a | -1.43 | -4.22 | -2.35 | -2.70 | -3.24 | -7.48 |
| *Bdnf* | 2.00 | -0.09 | -2.14 | -2.03 | -1.87 | -1.36 | *Smad2* | 0.77 | 1.22 | 1.08 | 0.89 | 1.16 | 0.56 |
| rno-miR-206 | 1.49 | 2.67 | 2.15 | 1.67 | 1.67 | 1.32 | rno-miR-18a | -4.15 | -3.48 | -4.45 | -4.64 | -4.02 | -4.17 |
| *Bcl2l11* | -2.85 | -0.11 | -2.85 | -0.78 | -1.47 | -2.85 | rno-miR-141 | -3.25 | -1.53 | -5.61 | -5.80 | -0.15 | -5.33 |
| rno-miR-17 | -0.16 | 0.41 | 1.12 | 1.28 | 1.83 | 1.66 | rno-miR-200a | -1.70 | -1.02 | -2.00 | -2.19 | -1.56 | -1.72 |
| *Cdkn1a* | 6.99 | 6.57 | 4.96 | 5.93 | 4.50 | 2.28 | *Smad4* | -0.06 | 0.59 | 0.06 | 0.75 | 0.44 | 0.29 |
| rno-miR-17 | -0.16 | 0.41 | 1.12 | 1.28 | 1.83 | 1.66 | rno-miR-18a | -4.15 | -3.48 | -4.45 | -4.64 | -4.02 | -4.17 |
| *Col1a1* | -1.10 | -2.65 | -1.34 | 1.27 | 2.97 | 2.28 | rno-miR-26a | -0.45 | 0.10 | 0.04 | -0.34 | -0.34 | 0.17 |
| rno-miR-196a | -4.04 | -3.63 | -7.00 | -7.19 | -6.56 | -6.72 | rno-miR-34a | -0.37 | -0.09 | 0.25 | -0.12 | 0.54 | 0.54 |
| *Ctgf* | 0.54 | 1.13 | 0.89 | 1.41 | 0.38 | 0.18 | rno-miR-146a | 0.09 | 0.00 | 1.71 | 1.93 | 3.52 | 3.15 |
| rno-miR-18a | -4.15 | -3.48 | -4.45 | -4.64 | -4.02 | -4.17 | *Smo* | 0.45 | 0.28 | 1.03 | 0.06 | 0.26 | -0.02 |
| rno-miR-30c | -0.44 | 0.07 | 0.30 | -0.10 | -0.03 | 0.28 | rno-miR-125b-5p | -0.72 | 0.00 | -0.63 | -0.82 | -0.20 | -0.35 |
| rno-miR-133a | -0.95 | -4.14 | -1.54 | -1.65 | -6.78 | -2.50 | rno-miR-324-5p | -1.65 | -2.09 | -0.93 | -2.01 | -1.45 | -1.18 |
| rno-miR-133b | -0.03 | -1.86 | -1.14 | -6.43 | -5.80 | -5.96 | rno-miR-326 | 0.19 | 1.47 | 1.22 | 0.59 | 0.90 | 0.76 |
| *Ctnnb1* | 0.10 | 0.18 | 0.39 | 0.74 | 0.80 | 0.15 | *Tgfbr2* | 0.81 | -0.51 | 1.49 | 1.52 | 2.42 | 0.72 |
| rno-miR-200a | -1.70 | -1.02 | -2.00 | -2.19 | -1.56 | -1.72 | rno-miR-21 | -0.18 | 1.13 | 2.62 | 2.48 | 3.07 | 2.65 |
| *Dll1* | -0.36 | -1.43 | -3.19 | -2.59 | 1.00 | -0.04 | rno-miR-133b | -0.03 | -1.86 | -1.14 | -6.43 | -5.80 | -5.96 |
| rno-miR-34a | -0.37 | -0.09 | 0.25 | -0.12 | 0.54 | 0.54 | rno-miR-211 | 0.20 | -3.19 | -1.85 | -1.60 | -6.37 | -6.53 |
| *Faslg* | 0.51 | 1.72 | 0.51 | 1.90 | 2.62 | 0.51 | *Tgfb1* | 2.24 | 2.68 | 2.86 | 3.62 | 4.48 | 3.45 |
| rno-miR-21 | -0.18 | 1.13 | 2.62 | 2.48 | 3.07 | 2.65 | rno-miR-133b | -0.03 | -1.86 | -1.14 | -6.43 | -5.80 | -5.96 |
| *Hoxb8* | 4.46 | 2.86 | 2.86 | 2.86 | 2.86 | 2.57 | *Tgfb2* | 0.25 | 1.61 | 1.06 | 0.14 | -0.13 | -0.04 |
| rno-miR-196a | -4.04 | -3.63 | -7.00 | -7.19 | -6.56 | -6.72 | rno-miR-141 | -3.25 | -1.53 | -5.61 | -5.80 | -0.15 | -5.33 |
| *Jag1* | 0.12 | 0.52 | 0.31 | 0.62 | 0.28 | -0.30 | rno-miR-200a | -1.70 | -1.02 | -2.00 | -2.19 | -1.56 | -1.72 |
| rno-miR-34a | -0.37 | -0.09 | 0.25 | -0.12 | 0.54 | 0.54 | *Tp53* | 1.11 | 1.40 | 1.29 | 1.61 | 1.64 | 0.99 |
| rno-miR-124 | -1.62 | -1.02 | -1.92 | -2.11 | -1.49 | -1.64 | rno-miR-34a | -0.37 | -0.09 | 0.25 | -0.12 | 0.54 | 0.54 |
| rno-miR-141 | -3.25 | -1.53 | -5.61 | -5.80 | -0.15 | -5.33 | *Wisp2* | 4.21 | 4.67 | 3.71 | 3.38 | 3.86 | 3.85 |
| *Notch1* | -0.16 | 0.18 | -0.18 | 0.44 | 0.61 | 0.08 | rno-miR-449a | -1.43 | -4.22 | -2.35 | -2.70 | -3.24 | -7.48 |
| rno-miR-146a | 0.09 | 0.00 | 1.71 | 1.93 | 3.52 | 3.15 | *Wnt1* | -1.70 | -2.87 | -3.85 | -2.37 | -1.74 | -3.29 |
| rno-miR-449a | -1.43 | -4.22 | -2.35 | -2.70 | -3.24 | -7.48 | rno-miR-21 | -0.18 | 1.13 | 2.62 | 2.48 | 3.07 | 2.65 |
| *Notch3* | 0.00 | 0.00 | 0.00 | 0.00 | 1.67 | 1.91 | rno-miR-34a | -0.37 | -0.09 | 0.25 | -0.12 | 0.54 | 0.54 |
| rno-miR-206 | 1.49 | 2.67 | 2.15 | 1.67 | 1.67 | 1.32 | *Wnt3* | 0.62 | -0.72 | -0.05 | 0.40 | -0.49 | -0.21 |
| *Pdcd4* | -0.14 | -0.33 | -0.35 | 0.29 | 0.73 | 0.67 | rno-miR-34a | -0.37 | -0.09 | 0.25 | -0.12 | 0.54 | 0.54 |
| rno-miR-21 | -0.18 | 1.13 | 2.62 | 2.48 | 3.07 | 2.65 | *Vsnl1* | -0.11 | -0.79 | -1.11 | -0.79 | -1.13 | -0.23 |
| *Pten* | 0.40 | 0.35 | 0.50 | 0.46 | 0.18 | 0.29 | rno-miR-290 | 0.48 | 2.00 | 1.87 | 1.33 | 1.39 | 1.05 |
| rno-miR-21 | -0.18 | 1.13 | 2.62 | 2.48 | 3.07 | 2.65 | *Irak1* | -0.12 | -0.32 | -0.25 | 0.01 | -0.12 | -0.26 |
| rno-miR-216a | 0.49 | 1.70 | -2.10 | -2.29 | -1.66 | -1.82 | rno-miR-146a | 0.09 | 0.00 | 1.71 | 1.93 | 3.52 | 3.15 |
| rno-miR-217 | -1.70 | 0.39 | -2.00 | -2.19 | -1.56 | -1.72 |  |  |  |  |  |  |  |
| *Reck* | -0.18 | -0.06 | -0.43 | -0.15 | -0.41 | -0.30 |  |  |  |  |  |  |  |
| rno-miR-21 | -0.18 | 1.13 | 2.62 | 2.48 | 3.07 | 2.65 |  |  |  |  |  |  |  |
